# Sex Specific Sleep Parameters Among People With Substance Use Disorder

**DOI:** 10.3389/fpsyt.2022.905332

**Published:** 2022-05-26

**Authors:** Caitlin E. Martin, Joseph M. Dzierzewski, Lori Keyser-Marcus, Emily K. Donovan, Tatiana Ramey, Dace S. Svikis, F. Gerard Moeller

**Affiliations:** ^1^Department of Obstetrics and Gynecology, School of Medicine, Virginia Commonwealth University, Richmond, VA, United States; ^2^Division of Therapeutics and Medical Consequences, Institute for Drug and Alcohol Studies, Virginia Commonwealth University, Richmond, VA, United States; ^3^Department of Psychology, Virginia Commonwealth University, Richmond, VA, United States; ^4^Department of Psychiatry, National Institute on Drug Abuse, Bethesda, MD, United States

**Keywords:** addiction, substance use disorder, opioid use disorder, sleep, cannabis use disorder, cocaine use disorder, sex differences

## Abstract

**Introduction:**

Sleep can have substantial impacts in substance use disorder (SUD) pathogenesis, treatment, and recovery. Sex differences exist in both sleep and SUD, but how sleep is uniquely associated with SUD by sex is not known. The study objective was to compare, within sex, sleep parameters between individuals with SUD and non-substance misusing controls.

**Methods:**

Secondary analyses of a parent cross-sectional study examining the feasibility and acceptability of a novel neurocognitive phenotyping assessment battery were completed. SUD and control subjects were recruited through local advertising and an established research registry. Subjects with SUD were also recruited through a university-based outpatient SUD treatment clinic. Self-reported sleep quality was assessed using the Pittsburgh Sleep Quality Index (PSQI). Sex-stratified *t*-tests compared sleep between SUD and control subjects while Crosstab analyses explored group differences in the proportion of individuals reporting poor sleep (defined as PSQI ≥ 5).

**Results:**

Data from 162 males (44 controls, 118 SUD) and 146 females (64 controls, 82 SUD) were included in the present study. For females only, a significantly lower proportion of controls reported PSQI-defined poor sleep than individuals with any SUD or specifically with opioid use disorder. Male, but not female, controls reported shorter sleep latency, longer sleep duration, and less sleep disturbance than males with each SUD type.

**Discussion/Implications:**

Sleep holds promise as an avenue to address SUD within a biopsychosocial model. Future work at the intersection of SUD and sleep should prioritize investigations of their interplay with sex to identify targets for tailored SUD interventions.

## Introduction

Deaths due to substance use disorder (SUD) occur more often in males than females, yet increases in SUD-related mortality are occurring more rapidly for females than males (1). Females with opioid use disorder (OUD) are witnessing faster increases in overdose rates due to fentanyl than males (2). Stimulant use has skyrocketed as a cause of death (3), with females burdening additional negative impacts (4, 5).

Sex differences exist in SUD risk (6, 7) and treatment (8, 9). Examples include females progressing more rapidly from initial use to SUD (10) and males receiving buprenorphine having worse OUD treatment continuation rates (11). Given these sex differences, achieving a deeper understanding of the role that sex, as a key biological variable, plays in SUD is warranted.

New, effective SUD treatment options tailored to individuals' neurobiological characteristics and social contexts are urgently needed (12). One area receiving increased attention as a target for SUD prevention, assessment, treatment and recovery is sleep (13). Sleep and SUD demonstrate a bi-directional relationship (14, 15), and this intersection likely brings complexity to SUD trajectories. Specifically, substance use itself can negatively impact sleep quality (16–18). Simultaneously, sleep health may heighten or buffer risk for SUD development (19) and treatment response (20–22). In the general population, sex differences exist across sleep parameters (23), with sleep disturbance generally being more common among females than males (24).

Prior efforts attempting to assess sex-specific associations between sleep and SUD have been limited and inconclusive (25). The present study's primary objective was to compare, within sex, sleep parameters between individuals with SUD and non-substance misusing controls. The secondary objective was to report sex-specific differences in sleep parameters by primary drug diagnosis between SUD and control subjects. We hypothesized that poor sleep parameters would be more prevalent among the SUD groups than controls for both sexes.

## Materials and Methods

The Virginia Commonwealth University IRB (IRB# HM 20012559) approved the study, and written informed consent was obtained.

### Subjects and Study Procedures

Methods for the parent study are described elsewhere (26). The objective of the parent, cross-sectional study was to assess the feasibility and acceptability of the National Institute on Drug Abuse (NIDA) Phenotyping Battery (PhAB), a novel package of self-report and neurobehavioral performance measures assembled by NIDA in consultation with an addiction expert workgroup. The PhAB is designed for eventual use in clinical trials to allow for classification of individuals with SUD along neurofunctional domains (e.g., behavioral phenotype), and to eliminate heavy reliance on DSM-5 criteria and primary drug of use to determine treatment strategies.

For the parent study, participants were recruited from an established patient registry, local advertising, and a SUD treatment clinic. Eligibility criteria were relaxed to recruit a heterogeneous sample of individuals with SUD along with non-substance misusing controls. Thus, individuals in the SUD group were not limited to be in a certain stage of recovery; active substance use was neither an inclusion nor an exclusion criterion. Inclusion criteria for both groups consisted of age between 18 and 70 years and ability to complete forms and interviews in English. Individuals enrolled in the SUD group also had to meet DSM-5 criteria for a current SUD with opioids, cannabis, and/or cocaine as the primary drug diagnosis. Conditions considered exclusionary were: current psychosis, mania, suicidal/ homicidal ideation, history of seizures (excluding childhood febrile seizures), or loss of consciousness from traumatic injury for more than 30 min, or any other illness, or condition, which in the opinion of the PI or study physician would preclude safe and/or successful completion of the study. Severe comorbid alcohol use disorder was exclusionary. Subjects meeting severe criteria for more than one drug (*n* = 5) were excluded from this secondary analysis. Non-substance misusing controls met the same criteria noted above, with the exception that they could not meet DSM-5 SUD criteria. All subjects were able to complete forms and interviews in English. At the study visit, subjects completed urine drug testing (UDT), questionnaires, and the PhAB measures.

### Measures

*The Pittsburgh Sleep Quality Index, PSQI* (27), a 19-item self-report tool, assessed overall sleep quality (range 0–21; higher scores indicate worse global sleep) along with seven component scores (range 0–3, higher scores indicate worse sleep). The PSQI is widely used to measure sleep difficulty. It has been validated in a range of settings and in a variety of samples, from children (28) to older adults (29). Based on prior validation studies, a total PSQI score ≥ 5 is associated with poor sleep quality (27).

*Demographic* information included age, sex (self-reported male vs. female), race, education, and employment status.

*Recent substance use* was determined via timeline follow-back interview (30) and UDT.

### Data Analysis

Analyses were conducted with SPSS version 26 (31) and stratified by sex. First, descriptive statistics were calculated for demographic and clinical characteristics. Continuous variables were summarized via means and standard deviations while categorical variables were summarized via counts and percentages. A series of *t*-tests were conducted comparing sleep characteristics (PSQI total score and component scores) between SUD and control subjects. Next, an additional set of *t*-tests were conducted comparing sleep characteristics between controls and SUD subjects by their primary drug diagnosis (e.g., cocaine, cannabis, and opioid). Lastly, a series of Crosstab analyses were used to investigate whether SUD and control subjects differed in the proportion with PSQI-defined poor sleep (i.e., PSQI ≥ 5).

## Results

Data were available for 162 males (44 controls, 118 SUD) and 146 females (64 controls, 82 SUD). Among male SUD subjects, about a third had a primary drug diagnosis for OUD (*n* = 53), followed by cocaine (*n* = 37) and cannabis (*n* = 28) use disorder. For female SUD subjects, OUD (*n* = 46) was the most common primary drug diagnosis followed by cannabis (*n* = 22) and cocaine (*n* = 14) use disorder. More SUD subjects identified as Black race (males 79%, females 73%) compared to controls (males 39%, females 39%; Table 1). Among SUD subjects, polysubstance use was common. For example, 45% of male OUD subjects reported past 30-day cocaine use with 34% (*n* = 18) having a UDT positive for cocaine. For female OUD subjects, 33% and 26% reported past 30-day cannabis and cocaine use, respectively, with 17% (*n* = 8) testing positive for each of these substances. A third of male and half of female OUD subjects were receiving medication treatment such as buprenorphine or methadone (data not shown).

**Table 1:** Demographic, clinical, and sleep characteristics of SUD and non-substance misusing control study participants.

	**Males (*****n*** **=** **162)**					**Females (*****n*** **=** **146)**				
	**Control** **(*n* = 44)**	**All SUD** **(*n* = 118)**	**Opioid UD** **(*n* = 53)**	**Cocaine UD ** **(*n* = 37)**	**Cannabis UD** **(*n* = 28)**	**Control** **(*n* = 64)**	**All SUD** **(*n* = 82)**	**Opioid UD** **(*n* = 46)**	**Cocaine UD** **(*n* = 14)**	**Cannabis UD ** **(*n* = 22)**
**Demographics**	M (SD)	M (SD)	M (SD)	M (SD)	M (SD)	M (SD)	M (SD)	M (SD)	M (SD)	M (SD)
Age	37.0 (15.8)	44.6 (12.4)	44.4 (10.4)	52.2 (9.4)	35.0 (12.7)	34.8 (13.7)	41.2 (13.1)	41.1 (12.5)	50.9 (6.6)	35.3 (14.1)
Race	N (%)	N (%)	N (%)	N (%)	N (%)	N (%)	N (%)	N (%)	N (%)	N (%)
Black/African American	17 (38.6)	93 (78.8)	39 (73.6)	37 (100.0)	17 (60.7)	25 (39.1)	60 (73.2)	34 (73.9)	12 (85.7)	14 (63.6)
White/Caucasian	22 (50.0)	20 (16.9)	112 (0.8)	0 (0.0)	9 (32.1)	26 (40.6)	18 (22.0)	11 (23.9)	1 (7.1)	6 (27.3)
All other races	5 (11.3)	4 (3.3)	2 (3.8)	0 (0.0)	2 (7.2)	12 (18.8)	3 (3.6)	1 (2.2)	1 (7.1)	1 (4.5)
Ethnicity										
Hispanic/LatinX	1 (2.3)	3 (2.5)	1 (1.9)	1 (2.7)	1 (3.6)	2 (3.1)	1 (1.2)	1 (2.2)	0 (0.0)	0 (0.0)
Non-hispanic/LatinX	40 (90.9)	108 (91.5)	50 (94.3)	33 (89.2)	25 (89.3)	59 (92.2)	75 (91.5)	43 (93.5)	12 (85.7)	20 (90.9)
Marital status										
Never married	26 (59.1)	58 (49.2)	24 (45.3)	16 (43.2)	18 (64.3)	36 (56.3)	50 (61.0)	28 (60.9)	6 (42.9)	16 (72.7)
Married/living with partner	12 (27.3)	32 (27.1)	17 (32.1)	10 (27.0)	5 (17.8)	21 (32.8)	17 (20.8)	10 (21.8)	3 (21.4)	4 (18.2)
Separated/divorced/Widowed	5 (11.4)	27 (22.9)	11 (20.7)	11 (29.7)	5 (17.9)	7 (10.9)	14 (17.0)	7 (15.2)	5 (35.7)	2 (9.1)
Past 30 days employment										
Full-time (35+hours/week)	16 (36.4)	40 (33.9)	20 (37.7)	7 (18.9)	13 (46.4)	24 (37.5)	18 (22.0)	12 (26.1)	2 (14.3)	4 (18.2)
Part time	8 (18.2)	22 (18.6)	9 (16.9)	6 (16.2)	7 (25.0)	15 (23.4)	8 (9.7)	3 (6.5)	1 (7.1)	4 (18.2)
Unemployed	5 (11.4)	37 (31.4)	19 (35.8)	15 (40.5)	3 (10.7)	3 (4.7)	36 (43.9)	21 (45.7)	8 (57.1)	7 (31.8)
Other	13 (29.5)	18 (15.2)	4 (7.5)	9 (24.3)	5 (17.8)	22 (34.4)	19 (23.2)	9 (19.5)	3 (21.4)	7 (31.7)
Education										
< High school	1 (2.3)	18 (15.3)	10 (18.9)	5 (13.5)	3 (10.7)	1 (1.6)	14 (17.1)	8 (17.4)	5 (35.7)	1 (4.5)
High school or GED	5 (11.4)	53 (44.9)	25 (47.2)	20 (54.1)	8 (28.6)	10 (15.6)	43 (52.4)	25 (54.3)	8 (57.7)	10 (45.5)
Some college	21 (47.7)	34 (28.8)	14 (26.4)	10 (27.0)	10 (35.7)	22 (34.4)	20 (24.4)	11 (23.9)	1 (7.1)	8 (36.4)
College degree or more	17 (38.6)	13 (11.0)	4 (7.5)	2 (5.4)	7 (25.0)	29 (45.3)	5 (6.1)	2 (4.3)	0 (0.0)	3 (13.6)
**Sleep**	M (SD)	M (SD)	M (SD)	M (SD)	M (SD)	M (SD)	M (SD)	M (SD)	M (SD)	M (SD)
PSQI global score (0–21)	5.11 (3.08)	6.92* (3.72)	7.62* (3.80)	5.62 (3.55)	7.36* (3.43)	5.66 (2.53)	7.27* (2.99)	7.63** (2.83)	6.21 (3.26)	7.19* (3.12)
Sleep quality (0–3)	0.86 (0.80)	1.28* (0.95)	1.40* (0.85)	1.14 (1.06)	1.25 (0.97)	1.19 (0.59)	1.41 (0.80)	1.37 (0.83)	1.50 (1.09)	1.43 (0.51)
Sleep latency (0–3)	0.73 (0.73)	1.25** (0.86)	1.37** (0.82)	1.11* (0.88)	1.21* (0.92)	0.91 (0.77)	1.26* (0.74)	1.35* (0.71)	1.21 (0.70)	1.10 (0.83)
Sleep duration (0–3)	0.52 (0.82)	1.24** (1.23)	1.17* (1.22)	1.35* (1.30)	1.21* (1.20)	0.64 (0.86)	1.04* (1.15)	1.13* (1.19)	0.86 (1.17)	0.95 (1.07)
Sleep efficiency (0–3)	1.41 (1.45)	1.15 (1.41)	1.37 (1.46)	0.41* (1.04)	1.75 (1.38)	0.95 (1.40)	1.20 (1.45)	1.35 (1.48)	0.64 (1.28)	1.24 (1.48)
Sleep disturbance (0–3)	0.82 (0.45)	1.12** (0.48)	1.21** (0.50)	1.03* (0.44)	1.07* (0.47)	0.97 (0.40)	1.15* (0.42)	1.17* (0.38)	1.14 (0.53)	1.10 (0.44)
Sleep medication (0–3)	0.14 (0.41)	0.29 (0.64)	0.44* (0.78)	0.11 (0.31)	0.25 (0.65)	0.17 (0.46)	0.56* (0.82)	0.67 (0.87)	0.29 (0.61)	0.48 (0.81)
Daytime dysfunction (0–3)	0.64 (0.65)	0.59 (0.70)	0.65 (0.71)	0.49 (0.69)	0.61 (0.69)	0.83 (0.72)	0.67 (0.74)	0.59 (0.72)	0.57 (0.65)	0.90 (0.83)

PSQI scores are missing for one male with opioid and one female with cannabis use disorder (UD). The * symbol indicates the differences between groups significant at p < 0.05.

The ** symbol indicates the differences between groups significant at p < 0.01.

In males, PSQI global scores were better among controls (*M* = 5.11, *SD* = 3.08) than in SUD subjects (*M* = 6.92, *SD* = 3.72), *t*_(159)_ = −2.88, *p* = 0.005. Male controls also had significantly lower PSQI global scores than males with primary drug diagnoses of OUD (*M* = 7.62, *SD* = 3.80) and cannabis use disorder (*M* = 7.36, *SD* = 3.43), *p* = 0.001, and 0.005, respectively. Generally, male controls reported statistically shorter sleep latency, longer sleep duration, and less sleep disturbances than SUD males with any primary drug diagnosis.

In females, PSQI global scores were better among controls (*M* = 5.66, *SD* = 2.53) than in SUD subjects (*M* = 7.27, *SD* = 2.99), *t*_(143)_ = −3.45, *p* = 0.001. Female controls also had significantly lower PSQI global scores than females with primary drug diagnoses of OUD (*M* = 7.63, *SD* = 2.83) and cannabis use disorder (*M* = 7.19, *SD* = 3.12), *p* < 0.001 and 0.05, respectively. Unlike their male counterparts, female controls did not report any PSQI component score that was statistically better across all primary drug diagnoses for SUD subjects. Refer to Table 1 for a complete listing of comparisons of PSQI-reported sleep across sexes and study groups.

**Figure 1:**
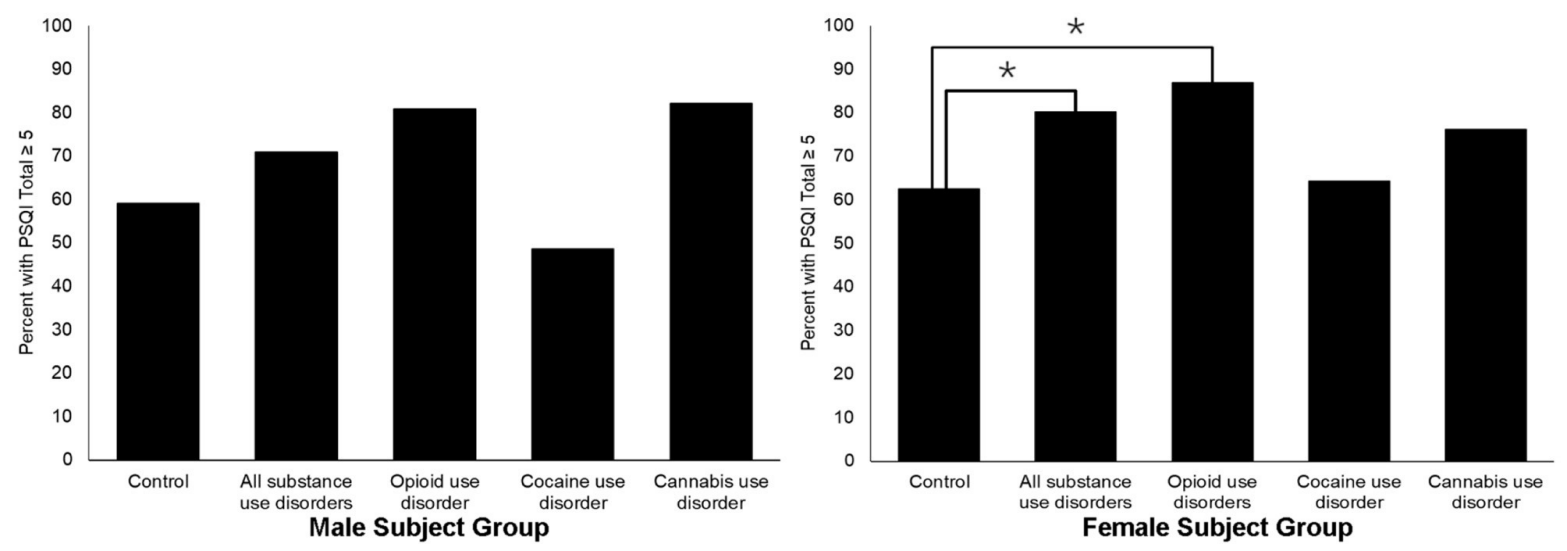
Sex-specific prevalence of PSQI score ≥5 for SUD and control study subjects. The *symbol indicates the differences between groups significant at *p* < 0.05.

Lastly, the proportion of males who reported PSQI-defined poor sleep did not differ between controls and SUD subjects, χ^2^ (1, *N* = 161) = 2.05, *p* > 0.05, nor primary drug diagnosis SUD subgroups. However, for females, a significantly lower proportion of controls reported PSQI-defined poor sleep than SUD subjects, χ^2^(1, *N* = 145) = 5.64, *p* < 0.05, or subjects with a primary drug diagnosis of OUD, χ^2^(3, *N* = 145) = 8.63, *p* <0.05. Refer to Figure 1 for a graphical depiction of group differences in the proportion of individuals with PSQI-defined poor sleep.

## Discussion

Sleep is an important component of health that can have widespread medical and psychosocial impacts (32). In our sample of individuals with SUD, we found poor sleep to be more prevalent compared to a control group for both males and females. These sleep differences were most notable for individuals with OUD and cannabis use disorder. However, only for females was overall poor sleep quality more prevalent among individuals with SUD compared to non-substance misusing controls.

In line with our hypotheses, PSQI scores indicated worse sleep quality among individuals with SUD compared to controls. This finding was expected given the emerging understanding of the bidirectional relationship between SUD and sleep (14). However, when we assessed differences from controls by primary drug diagnosis, consistent differences emerged for individuals with opioid and cannabis use disorder. These findings are consistent with literature highlighting the negative physiological effects opioid and cannabis use can have on sleep (18). More work is needed to better elucidate the underlying mechanisms of these associations within other clinical SUD populations.

The interplay between sleep and SUD is likely complex, resembling a co-existing comorbidity where precise functional interactions between sleep, sex, circadian rhythm, and other biological factors are unknown (13). Variation on an individual level could stem from both the specific substances being used and the biopsychosocial context (32), with many factors potentially related to both poor sleep and SUD progression. Our sample recruited from an outpatient clinic and its surrounding community were largely Black, underemployed, unmarried, and with low levels of completed education. This demographic snapshot reflects the high burden of social determinants of health common among many people with SUD, potentially reflecting social indicators of poverty and structural racism, which can also differentially impact sleep by one's gender (33). Importantly, social determinants of health play important roles in sleep health, and there is a call for further investigations into the mechanisms underlying disparities in sleep disruption using socio-ecological models (32, 33). Taken together, future research focused on sleep's intersection with SUD should incorporate multidimensional frameworks (34), tailored by sex and gender, in their study designs, analyses and interpretations.

When comparing control and SUD groups on clinically significant poor sleep quality (e.g., PSQI score of 5 or more), differences emerged for females only. Sleep disorders are more prevalent among females than males (35). Proposed underlying mechanisms for this disparity are numerous, from the role of sex-specific hormones (36) to social factors that more commonly impact females (33). However, the differential association of sleep and SUD presentation for females compared to males is novel. Our results indicate the importance of incorporating sex-stratified analyses into subsequent work aimed to better characterize the relationship between sleep and SUD.

The main limitation is the small sample size from a single site. This limitation precluded our ability to assess effect modification by sex in multivariable models, an area for future work. Further, age differed by sex and SUD groups, and will need to also be addressed in these investigations. The exclusion of subjects meeting DSM-5 severe criteria for alcohol use disorder may have limited generalizability, but doing so allowed us to focus on sex-specific associations between drug use disorders and sleep, an area lacking in research (37) more so than alcohol (38). Additionally, we did not examine study objectives by gender as gender identity was not assessed in the parent study. Gender influences risks for SUD (7) and poor sleep (33), and gender minority individuals are a high-risk population for SUD (39). Next, recruitment for the parent study was aimed at composing a “real world” SUD sample. This was a strength of the study. However, the SUD group varied widely in stages of recovery, from abstinence to active substance use. The sleep and SUD relationship is complex, stemming from a host of factors, including the direct effects of substance use (16). Future research at this intersection of sleep, SUD and sex should target SUD samples representing specific stages of treatment and recovery, such as individuals receiving medication for opioid use disorder. Lastly, our cross-sectional analyses prohibit conclusions regarding causality between sleep and SUD pathogenesis. Our results are intended to provide a foundation for future studies focused on identifying opportunities for targeting sleep as an avenue to mitigate harms related to SUD in a sex-informed way.

## Conclusion

Sleep problems and SUD substantially overlap neurobiologically as well as in their socio-ecological complexity. Sleep dysfunction and SUD differ by sex, as sex is one of the critical variables that shape an individual's overall health and daily functioning. Our results begin to shed light on the role of sleep dysfunction in SUD that needs to be addressed in a sex/gender-tailored way. Future work focused on the intersection of SUD and sleep should prioritize investigations of their interplay with sex, gender and social determinants of health to identify options for new SUD treatments specific to an individual as a part of his/her/their biopsychosocial profile.

## Data Availability Statement

The original contributions presented in the study are included in the article/supplementary material, further inquiries can be directed to the corresponding author.

## Ethics Statement

The studies involving human participants were reviewed and approved by the Virginia Commonwealth University IRB (IRB# HM 20012559). The patients/participants provided their written informed consent to participate in this study.

## Author Contributions

CM, JD, LK-M, FM, and DS conceptualized the manuscript. ED and JD performed data analysis. CM, JD, LK-M, ED, FM, and DS participated in interpretation of the results. CM and JD drafted the initial manuscript. TR, LK-M, ED, FM, and DS provided substantial revisions to the manuscript. FM and DS supervised the project. All authors reviewed and approved the final manuscript and the order of authors.

## Funding

This study was supported by National Institute on Drug Abuse (Bethesda, MD) grant U54DA038999 to FM. TR was substantially involved in U54DA038999 consistent with her role as Scientific Officer. She had no substantial involvement in the other cited grants. Other support includes: the National Institute on Aging of the National Institutes of Health award number K23AG049955 (PI: JD), National Institute on Drug Abuse award number K23DA053507 (PI: CM), and CTSA award number UL1TR002649 from the National Center for Advancing Translational Sciences (PI: FM).

## Author Disclaimer

The views and opinions expressed in this manuscript are those of the authors only and do not necessarily represent the views, official policy or position of the U.S. Department of Health and Human Services or any of its affiliated institutions or agencies.

## Conflict of Interest

The authors declare that the research was conducted in the absence of any commercial or financial relationships that could be construed as a potential conflict of interest.

## Publisher's Note

All claims expressed in this article are solely those of the authors and do not necessarily represent those of their affiliated organizations, or those of the publisher, the editors and the reviewers. Any product that may be evaluated in this article, or claim that may be made by its manufacturer, is not guaranteed or endorsed by the publisher.
